# Macrophage-derived CD36 + exosome subpopulations as novel biomarkers of *Candida albicans* infection

**DOI:** 10.1038/s41598-024-60032-7

**Published:** 2024-06-26

**Authors:** Shuo Li, Yanyan Xv, Yuanyuan Sun, Ziyi Shen, Ruiying Hao, Jingjing Yan, Mengru Liu, Zhao Liu, Tingting Jing, Xiaojing Li, Xiujuan Zhang

**Affiliations:** 1 Clinical Medical College of Hebei University of Engineering, Handan, 056000 China; 2 Department of Dermatology, Affiliated Hospital of Hebei University of Engineering, Handan, 056000 China; 3 Hebei Medical University, Shijiazhuang, 050000 China; 4 The Second Hospital of Hebei Medical University, Shijiazhuang, 050000 China; 5 Department of Laboratory, Affiliated Hospital of Hebei University of Engineering, Handan, 056000 China

**Keywords:** *C. albicans*, Exosomes, Immune escape, Extracellular traps, MPO, Nlrp3 inflammatory vesicles, CD36, PBA, Diagnostic markers, Skin diseases, Inflammation, Fungi

## Abstract

Invasive candidiasis (IC) is a notable healthcare-associated fungal infection, characterized by high morbidity, mortality, and substantial treatment costs. *Candida albicans* emerges as a principal pathogen in this context. Recent academic advancements have shed light on the critical role of exosomes in key biological processes, such as immune responses and antigen presentation. This burgeoning body of research underscores the potential of exosomes in the realm of medical diagnostics and therapeutics, particularly in relation to fungal infections like IC. The exploration of exosomal functions in the pathophysiology of IC not only enhances our understanding of the disease but also opens new avenues for innovative therapeutic interventions. In this investigation, we focus on exosomes (Exos) secreted by macrophages, both uninfected and those infected with *C. albicans*. Our objective is to extract and analyze these exosomes, delving into the nuances of their protein compositions and subgroups. To achieve this, we employ an innovative technique known as Proximity Barcoding Assay (PBA). This methodology is pivotal in our quest to identify novel biological targets, which could significantly enhance the diagnostic and therapeutic approaches for *C. albicans* infection. The comparative analysis of exosomal contents from these two distinct cellular states promises to yield insightful data, potentially leading to breakthroughs in understanding and treating this invasive fungal infection. In our study, we analyzed differentially expressed proteins in exosomes from macrophages and *C. albicans* -infected macrophages, focusing on proteins such as ACE2, CD36, CAV1, LAMP2, CD27, and MPO. We also examined exosome subpopulations, finding a dominant expression of MPO in the most prevalent subgroup, and a distinct expression of CD36 in cluster14. These findings are crucial for understanding the host response to *C. albicans* and may inform targeted diagnostic and therapeutic approaches. Our study leads us to infer that MPO and CD36 proteins may play roles in the immune escape mechanisms of *C. albicans*. Additionally, the CD36 exosome subpopulations, identified through our analysis, could serve as potential biomarkers and therapeutic targets for *C. albicans* infection. This insight opens new avenues for understanding the infection's pathology and developing targeted treatments.

## Introduction

*Candida albicans*, an opportunistic pathogenic fungus, typically resides as a commensal organism in the human body, colonizing mucosal surfaces such as the oral cavity, upper respiratory tract, gastrointestinal tract, reproductive tract, and skin without causing disease^1^. However, under conditions of immune system compromise or damage, Candidiasis poses a serious threat to human health. Upon infection, Toll-like receptors (TLRs) on host cells are activated, triggering innate immune responses. These TLRs, distributed across various cell types, initiate signaling cascades that release interleukins, chemokines, and proinflammatory substances, with interleukins playing a central role in modulating pro- and anti-inflammatory responses^2–4^. Macrophages, as key players in innate immunity, are the frontline defense against fungal infections, including those caused by *C. albicans* ^5^. The increasing incidence of invasive candidiasis (IC) underscores the clinical importance of early diagnosis, particularly in perioperative patients. Traditional fungal culture methods are time-consuming and often inadequate for early diagnosis. Molecular biology techniques, while detailed, are cumbersome and expensive. Existing biomarkers, such as Candida spp. Mannan, anti-mannan antibodies, and β-D-glucan, often lack specificity for invasive candidiasis^6,7^. Consequently, identifying new biomarkers for *C. albicans* infections is of paramount importance.

Extracellular vesicles (EVs), membranous structures released by both eukaryotic and prokaryotic cells, are present in various bodily fluids like blood, breast milk, urine, semen, and saliva^8–10^. EVs are formed through mechanisms involving the ESCRT complex, tetraspanins, ceramide-producing sphingomyelinases, phospholipid repositioning, and actin cytoskeleton depolymerization, although these processes are not fully understood^8^. EVs are broadly categorized into apoptotic vesicles, microvesicles, microparticles, and exosomes (Exos), liquid and extracellular components such as proteins, lipids, metabolites, and small molecules can enter the cell through endocytosis of the plasma membrane together with cell surface proteins to form early sorting endosome (ESE), which matures to form late sorting endosome (LSE), which secondarily invaginates to form intralumenal vesicles (ILVs), and eventually produces intralumenal vesicles (ILVs) containing multiple ILVs. After maturation, LSE is formed, and the secondary invagination of LSE forms ILVs, which eventually give rise to multivesicular bodies (MVBs) containing multiple ILVs, which fuse with the plasma membrane to release ILVs, i.e., exocytosis. MVBs fuse with the plasma membrane to release ILVs, i.e., Exosomes, while other EVs can be released directly from the plasma membrane^9^.

Exosomes, with diameters ranging from 30 to 150 nm, play crucial roles in intracellular signaling, inflammation, antigen presentation, apoptosis, and homeostasis^11^. Their potential in immunomodulation makes them promising carriers for therapeutic drugs across various diseases^12^. In addition, the molecular composition of exosomes is highly heterogeneous due to different stimuli in the microenvironment and cellular origins. Its surface membrane proteins derived from primitive cells can reflect the physiological, pathological as well as functional status of their primitive cells, and have great potential as disease biomarkers in the future. It has been demonstrated in a number of diseases, including cardiovascular diseases^13^, central nervous system-related diseases^14^, cancer^15^, liver disease^16^, kidney disease^17^ etc. However, some exosome surface protein markers are so low in abundance that they cannot be detected in samples. Recently, a new single exosome analysis technique with high sensitivity and good specificity has been reported to identify the surface protein composition of a single exosome by antibody DNA concatenation and high-throughput sequencing, thus extending the study of exosome heterogeneity^18^.

In this study, we extracted exosomes from THP-1 cells and *C. albicans* (SC5314)-infected THP-1 cells. We employed the proximity barcoding assay (PBA), a novel method offering high-throughput, single-particle, multi-protein detection of exosomes^18^. This approach aims to uncover new biomarkers for *C. albicans* infection, thereby contributing to the early diagnosis and effective management of this increasingly prevalent fungal disease.

## Results

The principal findings of this investigation are comprehensively delineated in Table 1

**Table 1:** The principal findings of this investigation.

Parameter	MPO in *C. albicans* immune evasion	CD36 as biomarker for *C. albicans*
Function/role	Myeloperoxidase (MPO) expression in macrophage-derived exosomes during *C. albicanss* infection	CD36 expression as a pattern recognition receptor in macrophage-derived exosomal subpopulations
Key observations	Enhanced MPO expression in exosomes from *C. albicans*-infected macrophages < br >—Association with NETosis and METs formation < br >—Contribution to immune evasion by *C. albicans*	Differential CD36 expression in response to *C. albicans* < br >—Potential role in cellular pyroptosis and immune response modulation
Findings	Significantly increased MPO in infected versus uninfected macrophages. < br > Elevated METs release under PMA and *C. albicans* stimulation	Notably higher CD36 levels in exosomal subpopulations during *C. albicans* infection
Clinical Implications	MPO as a novel target for elucidating *C. albicans* immune evasion strategies	CD36 subpopulations as promising early biomarkers for *C. albicans* infection; potential therapeutic targets in fungal infections

### Differentiation of Thp-1 cells into M0 macrophages detected by high content and q-PCR

**Figure 1:**
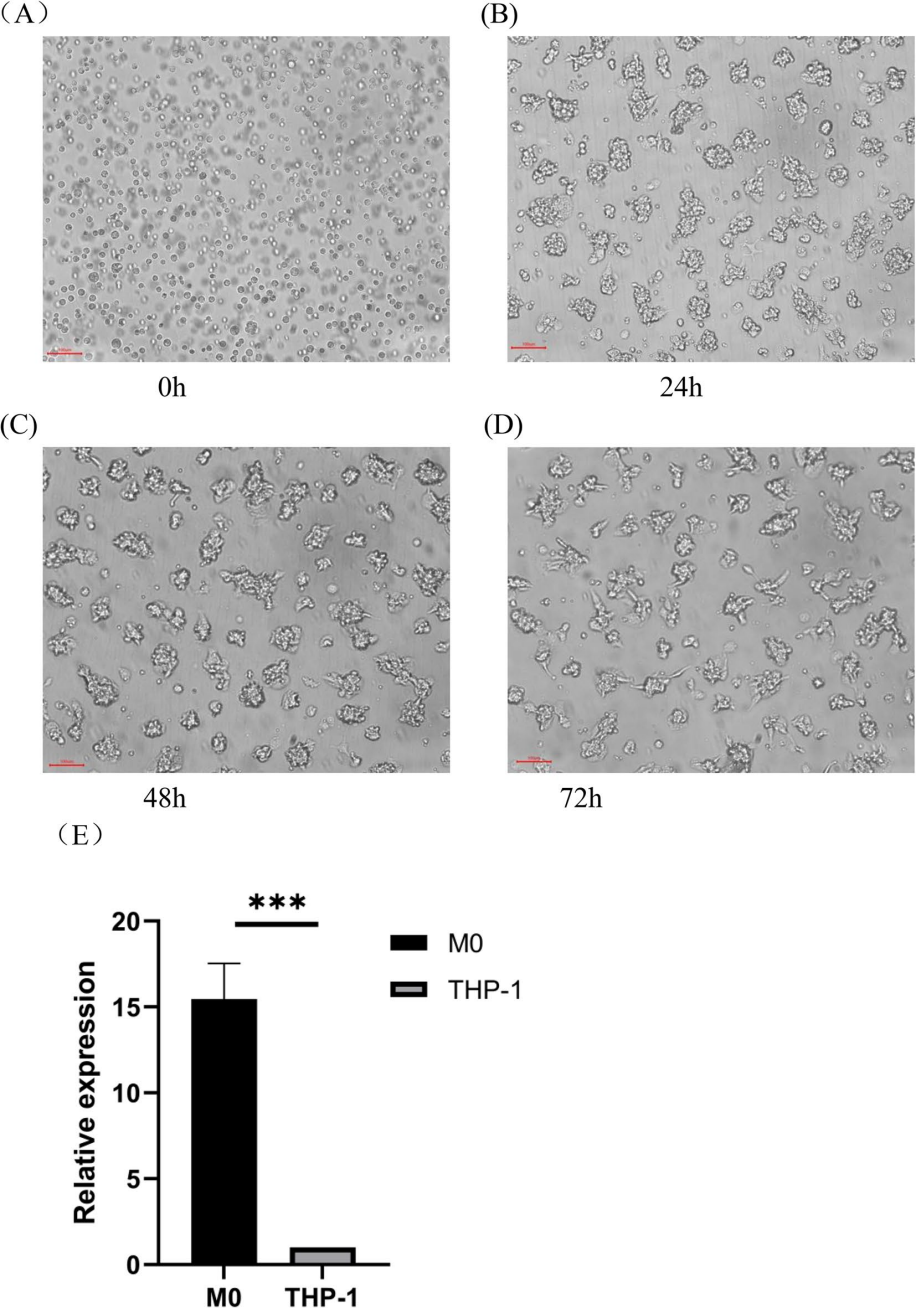
PMA induced THP-1 cells as M0 macrophages(100 $\times$). (**A**)THP-1 cells were induced by PMA after 0 h, shown under microscope. (**B**) THP-1 cells were induced by PMA after 24 h, shown under microscope. (**C**) THP-1 cells after 48 h of induction by PMA, shown under microscope. (**D**) THP-1 cells after 72 h of induction by PMA, shown under microscope. (**E**) PCR detection of the M0 macrophage surface marker CD11b.

The induction of THP-1 cells into M0-type macrophages was observed post-treatment with 75 ng/ml PMA for 72 h. Morphological changes, evident in Fig. 1A–D, included a transition from smaller, rounded suspended cells to larger, spindle-shaped, wall-adherent cells with multiple pseudopods and protrusions. An increase in cell volume correlating with stimulation time was noted, indicative of successful differentiation into M0-type macrophages.

The upregulation of CD11b, a surface marker for M0-type macrophages, was confirmed via q-PCR. Figure 1E shows a 15.24-fold increase in CD11b expression following 72 h of PMA treatment, further validating the differentiation of THP-1 cells into M0-type macrophages.

### Macrophage infection by *C. albicans* under high internalization

**Figure 2:**
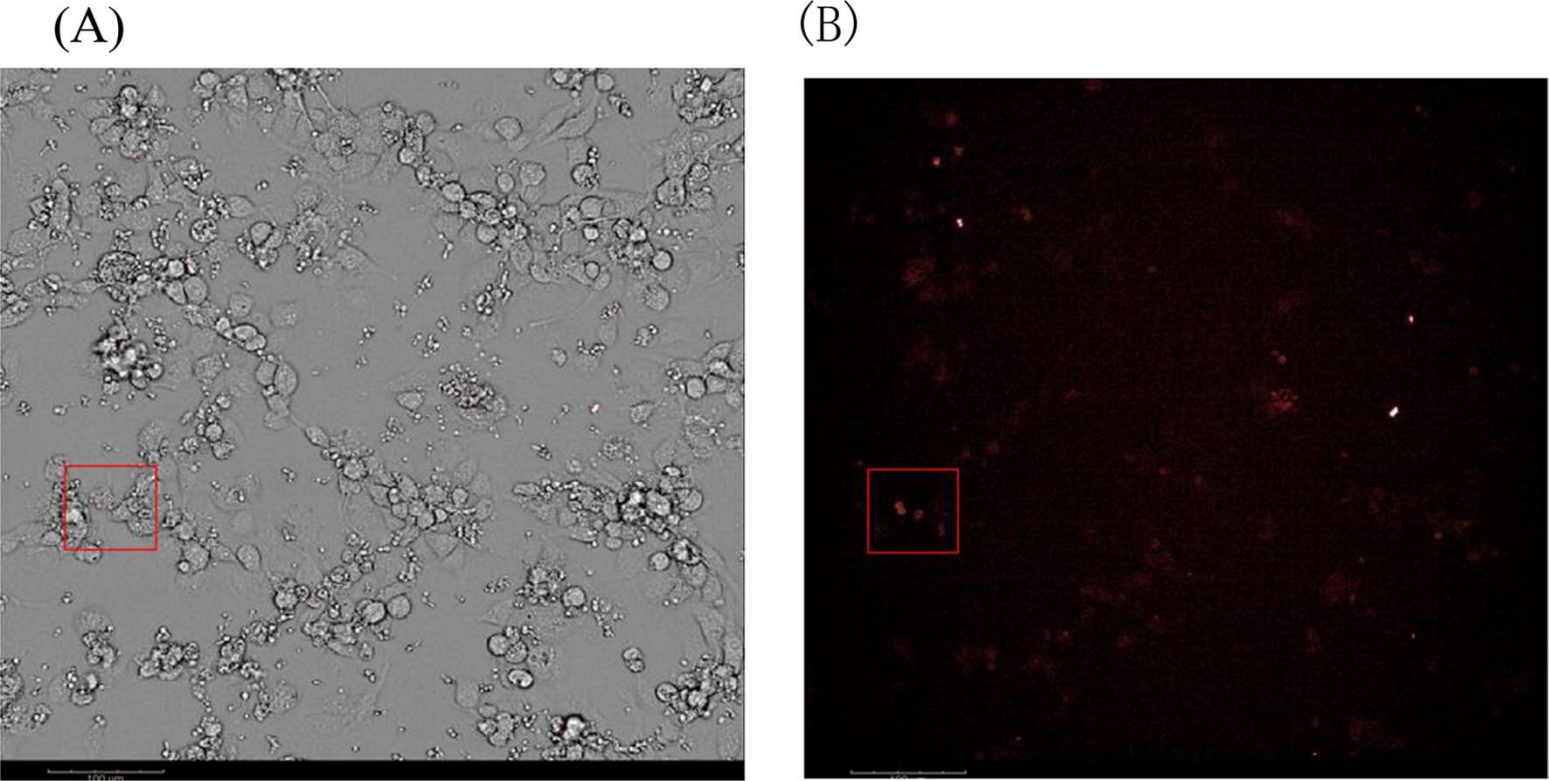
Macrophage phagocytosis of *C. albicanss* at 2.5 h under high content. (**A**) Under bright light, macrophages phagocytose *C. albicans*. (**B**) Under fluorescence, macrophage phagocytosis of *C. albicans*.

*C. albicans* was labeled using pHrodo Deep Red, a pH sensor dye that fluoresces in acidic environments. This facilitated the distinction between phagocytosed and unphagocytosed cells. Figure 2 displays the fluorescent signal indicative of macrophages having phagocytosed *C. albicans.*

### Exosome purification and characterization

**Figure 3:**
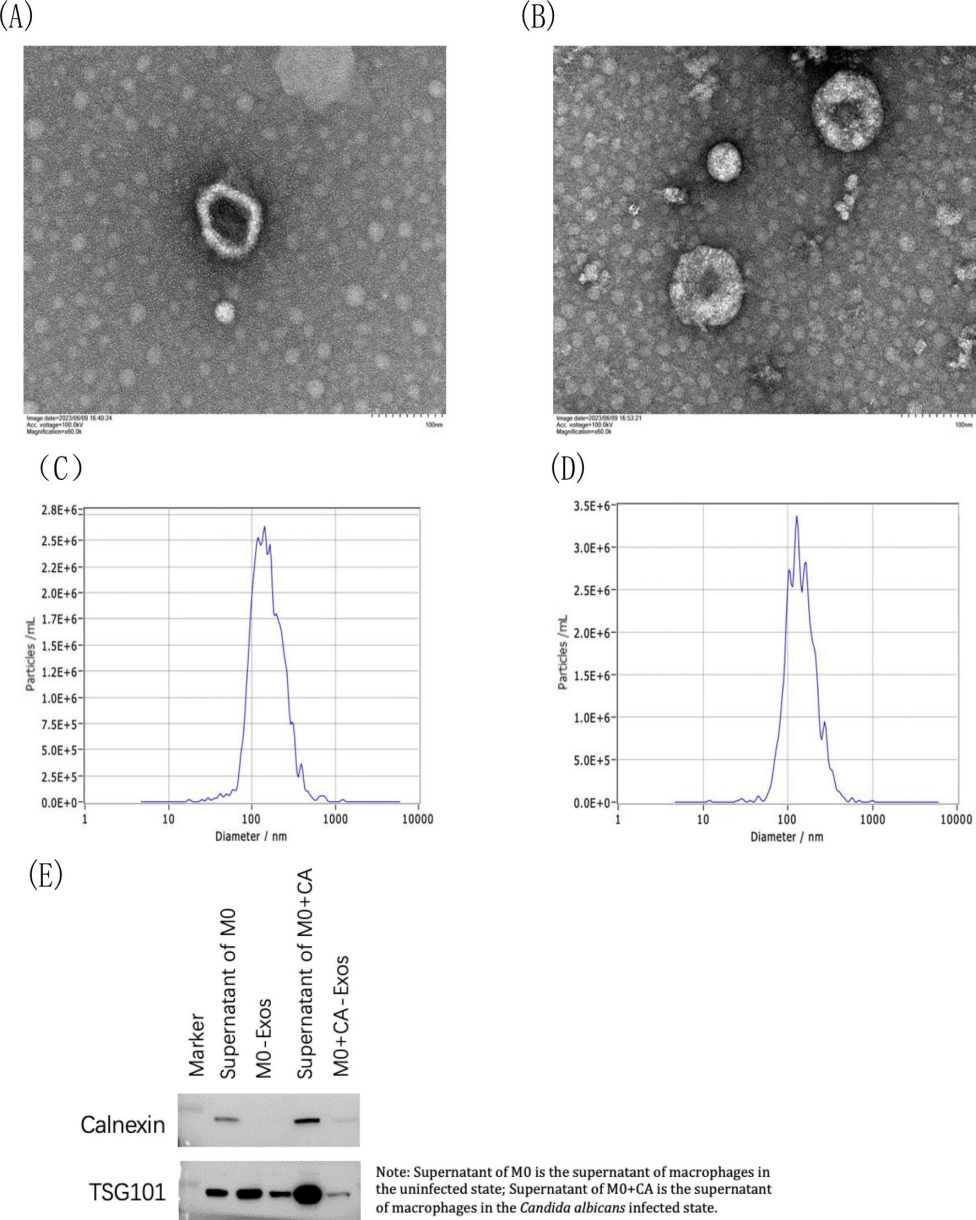
Characterization of two groups of exosomes. (**A**) TEM images of M0 group exosomes. Scale bar = 100 nm, (**B**) TEM images of exosomes in M0 + CA group. Scale bar = 100 nm. (**C**) NTA analysis of the size and distribution of exosomes in group M0. (**D**) NTA analysis of the size and distribution of exosomes in M0 + CA group. (**E**) Expression of the exosome marker TSG101 and no expression of the negative marker protein Calnexin were detected by western blot Supplementary Information.

Post-extraction of exosomes from both groups via ultracentrifugation, TEM, NTA, BCA, and western blotting were employed for characterization before the PBA assay. TEM images (Fig. 3A,B) revealed vesicle-like structures with a lipid bilayer, typical of exosomes. NTA analysis (Fig. 3C,D) determined the average particle sizes to be approximately 91 nm and 100 nm for the M0 and MO + CA groups, respectively. Western blotting confirmed the presence of the exosome marker TSG101 and the absence of the negative marker protein Calnexin^19^, indicating successful exosome extraction.

### PBA detection of exosome surface proteins

**Figure 4:**
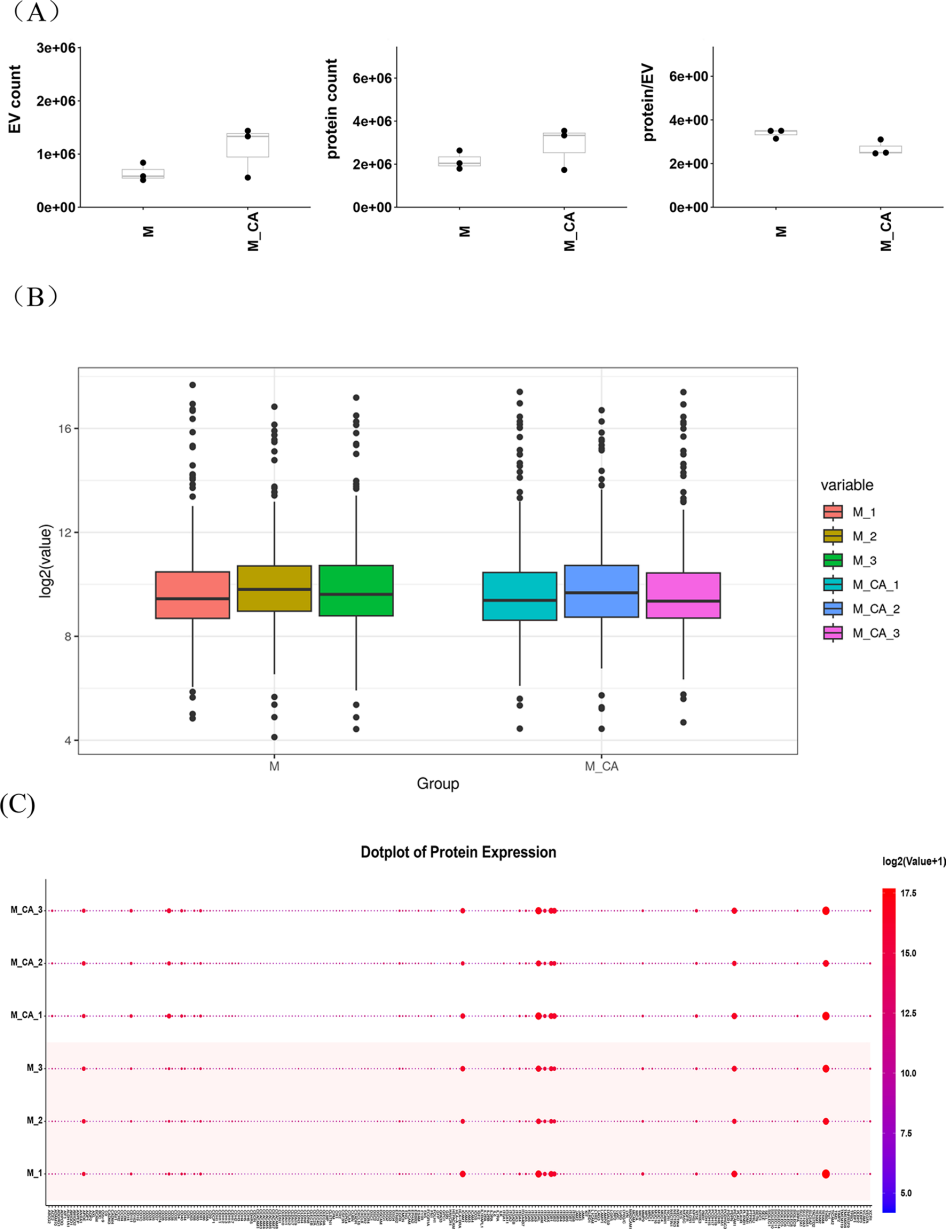

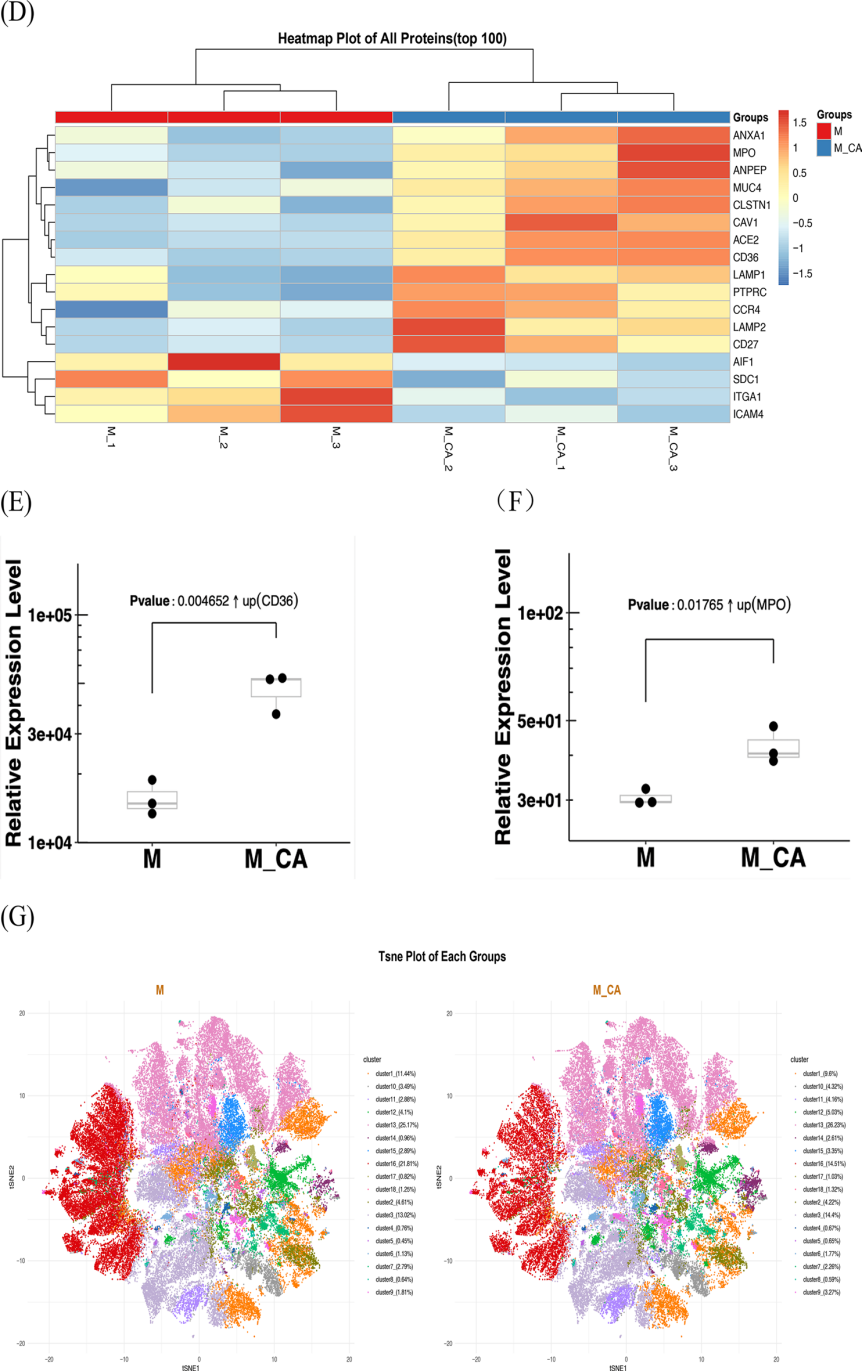

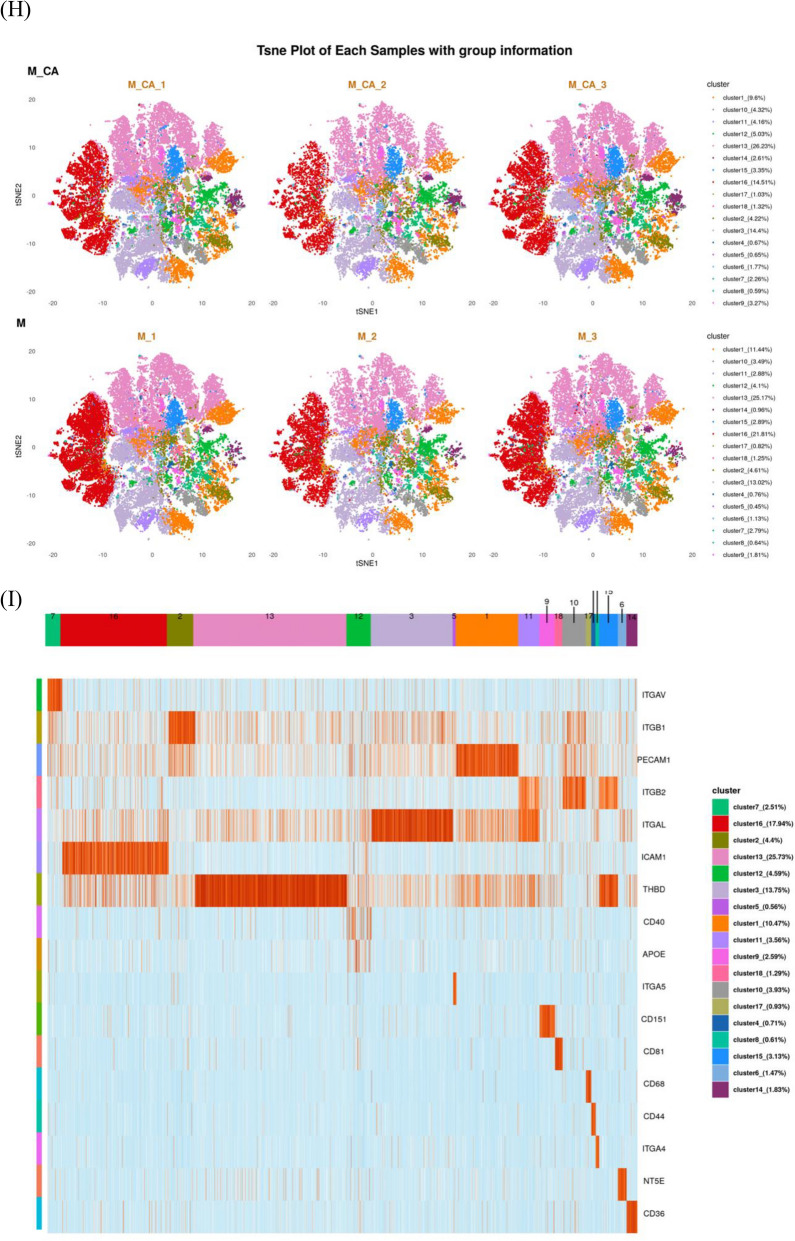

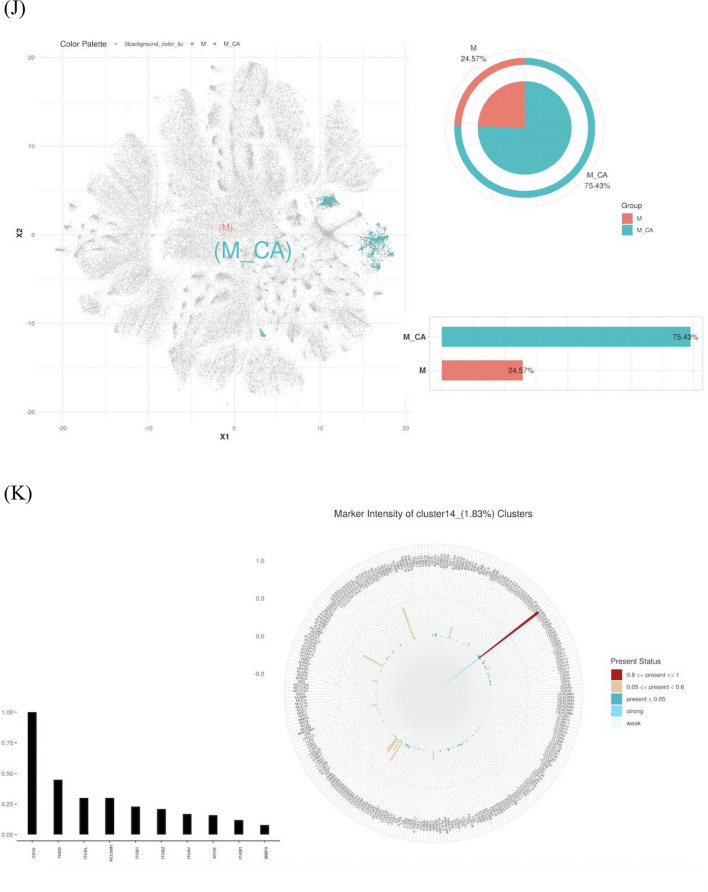
Identification and characterization of exosome subpopulations. (**A**) Number of exosomes detected, number of proteins, and number of proteins per exosome for both sets of samples. (**B**, **C**) Protein expression on the surface of exosomes in each sample. (**D**) Proteins differentially expressed by exosomes in the M0 and MO + CA groups. (E) CD36 expression was higher in the M0 + CA group, **P < 0.01. (**F**) MPO expression was higher in the M0 + CA group, *P < 0.05. (**G**) Distribution and proportion of exosome subpopulations in the M0 and MO + CA groups. (**H**) Distribution and proportion of exosome subpopulations in each sample. (**I**) Protein expression of each exosome subpopulation. (**J**) Distribution and proportion of cluster14 in the M0 and MO + CA groups, respectively. (**K**) Protein expression of cluster14.

PBA was utilized to evaluate individual EVs and their protein expressions in both sample groups (Fig. 4A). No significant differences were observed between the groups. Post-normalization using the TMM method, Fig. 4B,C illustrates the protein expression levels, highlighting the proteins with significant differential expression in the heatmap (Fig. 4D). Thirteen proteins were upregulated, and four downregulated in the MO + CA group, with notable increases in ACE2, CD36, CAV1, LAMP2, CD27, and MPO, and decreases in ITGA1, ICAM4, SDC1, and AIF1. Significant elevations in CD36 and MPO were observed in the MO + CA group compared to the M0 group (Figs. 4E,F).

FlowSOM analysis of single exosomes identified distinct subgroups based on exosomal protein characteristics. The distribution and proportion of all subgroups within two groups and each of their samples were visualized using t-SNE dimensionality reduction (Fig. 4G,H). The situation of proteins that are more highly expressed in each subgroup is depicted in Fig. 4I. Notably, Cluster14, although representing a minor proportion in the M0 group (0.96% as shown in Fig. 4G), was significantly increased to 2.61% in the M0 + CA group. Within Cluster14, 75.43% originated from the M0 + CA group. Cluster14 was then distinctly highlighted in the t-SNE plot (Fig. 4J) for an in-depth analysis of protein expression within this cluster, leading to the generation of a chart depicting the detection frequency of each protein in the subgroup (Fig. 4K). This analysis revealed a relatively higher expression of CD36. Consequently, we have designated Cluster14 as the CD36 subpopulation for further investigation.

## Discussion

Our data were indexed using bcl2fastq (Illumina) to convert the BCL file for each sample into fastq format and paired indexes. The complexTag, proteinTag, and moleculeTag were then extracted from the three fixed segments of each read. reads with only one count were excluded from the analysis. An in-house developed Perl script was then used to sequentially sort the tags based on complexTag, proteinTag, and moleculeTag. The number of exosomes with a particular protein combination was counted using an in-house developed R script. The applied t-SNE algorithm was implemented in the R package Rtsne with all parameters set to default values in our pipeline.

Recent studies increasingly underscore the significance of exosomes in both biophysiological and pathological processes, highlighting their evolving roles in diagnostics and therapeutics across a spectrum of diseases^20–23^. Furthermore, the exosomes secreted by host cells in response to fungal infections have garnered considerable attention in the scientific community, becoming a focal point of extensive research^24,25^. This growing body of evidence reflects the expanding understanding of exosomal functions and their potential implications in disease management and treatment strategies.

### MPO as a key factor in the immune evasion of *C. albicans*

In the context of increasing use of immunosuppressants, broad-spectrum antibiotics, and invasive procedures, the incidence of invasive fungal infections has risen significantly^26^. Among these, Candida species are predominant causative agents, with *C. albicans* being a common clinical species and a notable source of hospital-acquired infections^27^ Upon infection, *C. albicans* initiates immune responses through the recognition of its pathogen-associated molecular patterns (PAMPs) by host pattern recognition receptors (PRRs). Endocytosis mediated by endothelial cells via N-cadherin plays a role in the internalization of *C. albicans* ^28^, which can subsequently escape through vesicle phagocytosis or endothelial cell loss^29^. Then phagocytes, integral to the innate immune system, migrate from blood vessels to accumulate at sites of *C. albicans* infection^30^. This group primarily includes monocytes, macrophages, and neutrophils. Macrophages play a pivotal role in inhibiting and destroying *C. albicans* through mechanisms like nutrient deprivation, low pH environments, and oxidative stress^31^. Additionally, macrophages secrete immune factors that recruit more macrophages for the clearance of *C. albicans*^32^. However, *C. albicans* has developed various strategies to evade immune detection, diminish the effectiveness of antimicrobial responses, and escape from immune cells post-phagocytosis, though the specific mechanisms remain unclear^33^. Our study reveals that exosomes secreted by macrophages infected with *C. albicans* show a significant increase in myeloperoxidase (MPO) expression compared to those from uninfected macrophages. MPO, a key enzyme secreted by neutrophils and macrophages, is linked to the severity and prognosis of various cardiovascular diseases^34^ and is associated with NETosis, a form of cell death inducing the formation of extracellular traps (ETs)^35^. ETs, primarily identified in neutrophils as neutrophil extracellular traps (NETs), comprise components like histones, cathepsin G, neutrophil elastase, MPO, and others, crucial for pathogen capture and clearance^36^, are structures with bactericidal functions that occur by neutrophils stimulated by PMA, endotoxin, etc., and are known as neutrophil extracellular traps (NETs). The main components of ETs include histones (H1, H2A, H2B, H3, and H4), cathepsin G (CG), neutrophil elastase (NE), MPO, calcineurin, lactoferrin, and gelatinase^37^. These components are important for facilitating pathogen capture and clearance, with NE, MPO, and H3 being the major antimicrobial component proteins in NETs. The release of ETs is not limited to neutrophils but can also be released when other immune cells are exposed to different stimuli, including macrophages, mast cells, and others. In this paper, after THP-1 cells formed M0 macrophages after PMA stimulation, the exosomes produced by them expressed MPO, which may indicate that macrophages have macrophage extracellular traps (METs) released under PMA stimulation, while the exosomes produced by M0 macrophages after *C. albicans* infection expressed MPO was significantly increased, which may suggest that macrophages have increased release of METs under the dual stimulation of PMA and *C. albicans*. Bacterial and Fungal Infections Significantly Increased When MPO and NE Genes Were Knocked Out in Mice^38^, and it has been demonstrated that the formation of METs provides an immune escape route for *C. albicans* ^39^. Thus, MPO emerges as a potential target for understanding and combating *C. albicans* immune escape mechanisms.

### CD36 subpopulations as a potential biomarker for *C. albicans* Infection

CD36, a widely expressed scavenger receptor on various immune and non-immune cells, mediates numerous biological processes including inflammation, angiogenesis, atherosclerosis, and innate immunity^40,41^. Acting as a pattern recognition receptor (PRR) on macrophages, CD36 is involved in the phagocytosis and elimination of pathogens^42^. Additionally, CD36 recognizes endogenous ligands such as thrombospondin-1 (TSP-1), amyloid, advanced glycation end-products (AGEs), and advanced oxidation protein products (AOPPs)^43^. These ligands, indicative of cellular oxidative stress and lipid or protein denaturation, upon binding to CD36, trigger several pathophysiological responses, including inflammation and intracellular lipid accumulation^44,45^. Given its multifunctionality, CD36 is a promising biomarker for numerous diseases.

In the realm of gene expression, CD36 can be modulated by various environmental stimuli, transcription, post-translational modifications, or translocation to the plasma membrane^41^. Our study revealed a significant differential expression of CD36 in exosomal subpopulations between two groups, with a notably high expression level of CD36. This observation indicates that CD36 subpopulations may serve as viable biomarkers for the early detection of *C. albicans* infection, a hypothesis that merits further investigation and validation through future studies utilizing animal models. Moreover, CD36 may be implicated in another form of immune escape by *C. albicans*, known as cellular pyroptosis. Following phagocytosis by macrophages, *C. albicans* transforms within phagosomes from yeast to mycelium morphology. When mycelium growth within the phagosomes exceeds the phagosomal membrane's capacity, the membrane ruptures, activating the host's Nlrp3 inflammasome^46^. The structural interaction between receptor proteins and their ligands serves as a bridge, connecting receptor and effector proteins, which induces the activation of the inflammatory protease caspase-1. Caspase-1 cleaves gasdermin D, which then binds to phospholipoproteins on the cell membrane, forming pores and releasing a significant amount of pro-inflammatory factors. This cascade results in cell lysis and pyroptosis^39^. Notably, in atherosclerosis^47^, CD36 has been identified to play a dual role in both the initiation and activation of Nlrp3 inflammasomes, a hallmark of cellular pyroptosis. While the precise mechanism remains to be fully elucidated, it is plausible that CD36 also plays a crucial role in mediating immune responses following *C. albicans* infection in macrophages. Consequently, CD36 emerges not only as a promising biomarker for *C. albicans* infection but also as a potential target for therapeutic intervention in fungal infections and associated inflammatory responses.

## Conclusion

In conclusion, employing the PBA approach enabled us to analyze millions of individual exosomes per sample, facilitating the screening for biomarkers of *C. albicans* infection at the level of individual exosomes. Our findings suggest that Myeloperoxidase (MPO) may play a role in the immune escape mechanisms of *C. albicans*, and that CD36 holds potential as a biomarker for detecting such infections. We observed that *C. albicans* infection alters the surface protein profiles of exosomes in both study groups. These alterations could represent a compensatory mechanism that promotes inflammatory and immune responses, thereby enriching our understanding of the immune mechanisms involved in *C. albicans* infections. Furthermore, to validate these early diagnostic markers and therapeutic targets, and to confirm the functional roles of MPO and CD36, multicenter studies involving both animal models and patient populations are essential. Future research should focus on elucidating the molecular mechanisms of MPO and CD36, which will deepen our understanding of their roles in *C. albicans* infections.

## Materials and methods

The authors confirm that the ethical policies of the journal, as noted on the journal’s author guidelines page, have been adhered to. No ethical approval was required as the research in this article related to micro-organisms.

**Figure 5:**
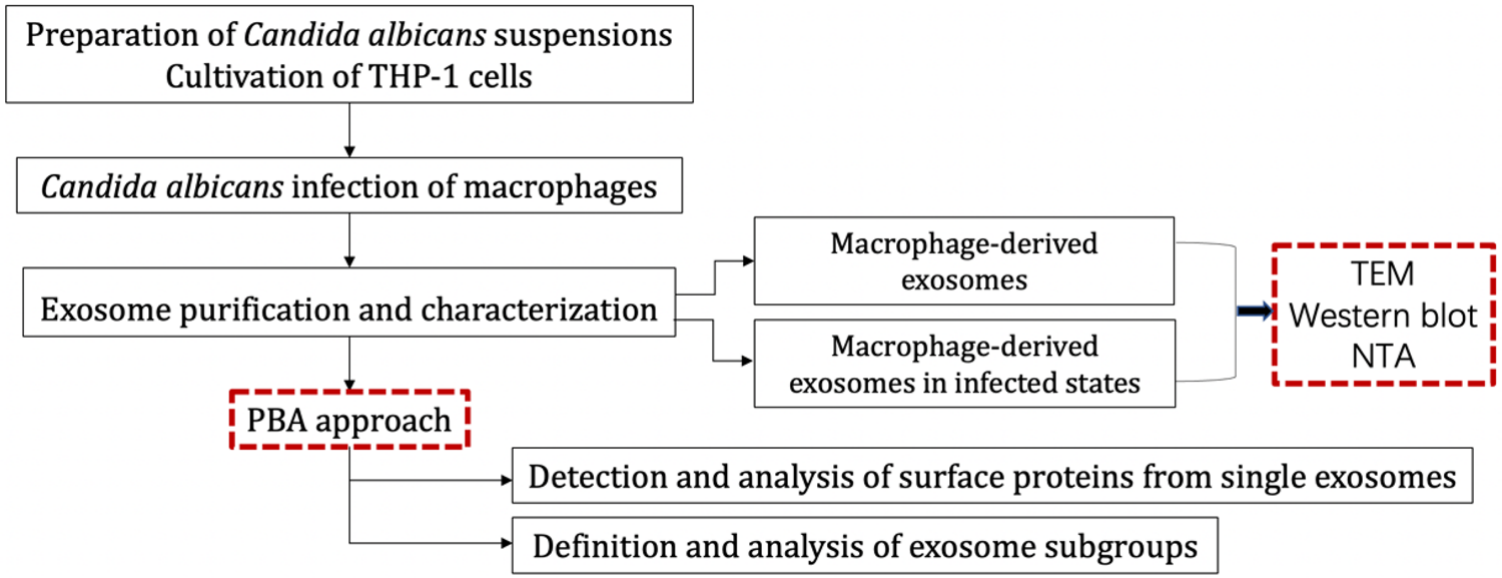
Flow chart of this study.

The flowchart of this paper is shown in Fig. 5.

### Strain activation and suspension preparation

*C. albicans* SC5314 (a kind gift from Shanghai Changzheng Hospital) and THP-1 cells (purchased from Procell, Item No. CL-0233) were used in this study. *C. albicans* SC5314 bacterial fluids, thawed at room temperature from frozen storage tubes, were inoculated into 5 ml of YPD liquid medium in a clean room biosafety cabinet. The culture was maintained at 37 °C on a shaker at 150 rpm overnight to ensure the strain reached the logarithmic growth phase. The culture was then centrifuged at 1500 rpm for 10 min; the supernatant was discarded, and the cells were washed twice with PBS. A 0.9% sodium chloride solution was used to adjust the bacterial solution concentration to 2 × 10^8^ CFU/mL^48^.

### Cell culture and induction

Frozen THP-1 cell lines were resuscitated in T300 culture flasks at 37 °C in a humid environment containing 5% CO_2_ using RPMI-1640 medium (containing 1% double antibody, 10% fetal bovine serum, and 0.05 mM β-mercaptoethanol)^49^. Upon reaching a cell density of 8 × 10^5^ cells/ml, the cells were centrifuged at 1000 rpm for 5 min, and the medium was replaced with fresh THP-1 complete medium. Cells were then distributed into 6-well plates at 2 mL per well, and Phorbol-12-Myristate-13-Acetate (PMA) was added to achieve a concentration of 75 ng/ml^50^. Cells were incubated at various time points (0 h, 24 h, 48 h, 72 h) for high internal culture and imaging.

### q-PCR to detect Thp-1 cell differentiation

RNA was extracted using TRIzol reagent, and the total RNA content was measured using a nucleic acid quantifier. Reverse transcription was performed following the instructions of the Reverse Transcription Kit (Item No.7E652J2, Novozymes). The q-PCR reaction conditions were set at 37 °C for 45 min, followed by 85 °C for 5 s. The reaction products were preserved for subsequent experiments. q-PCR was performed according to the Realtime PCR Master Mix Kit (SYBR Green Realtime PCR Master Kit, Toyobo), with GAPDH as the internal reference. The primer sequences ^51^are shown in Table 2:

**Table 2:** PCR primers for CD11b and GAPDH.

sequences	sequences(5′ to 3′)
GAPDH-F	GGAGCGAGATCCCTCCAAAAT
GAPDH-R	GGCTGTTGTCATACTTCTCATGG
CD11b-F	GCCTTGACCTTATCTCATGGG
CD11b-R	CCTGTGCTGTAGTCGCACT

### *C. albicans* stain

A 1 mL sample of *C. albicans* (2 × 10^8^ CFU/ml) was centrifuged and the supernatant discarded; the cells were washed twice with wash solution and then centrifuged (500 g, 5 min, 4 °C). The cells were resuspended in 1 mL of wash solution and added to pHrodo Deep Red stain^52^, shaken until dissolved, and incubated at 22.5 °C for 2 h. After adding 1 mL of DB medium to resuscitate the fungus, the cells were centrifuged again to discard the supernatant. The cells were washed once with RPMI-1640, centrifuged, and then resuspended in 1 mL of RPMI-1640, adjusting the bacterial concentration accordingly.

### Highly visceral record of infection

Stained *C. albicans* was uniformly added to the THP-1 cell culture medium at a ratio of 1 fungus per macrophage, and the cells were placed under high internalization for observation of phagocytosis.

### Exosome purification and characterization

In this study, research subjects were allocated into two distinct groups for analytical purposes: the negative control group consisted of subjects with macrophage-derived exosomes in a non-infected state; conversely, the positive control group comprised subjects exhibiting macrophage-derived exosomes subsequent to infection with *C. albicans*.

THP-1 cells were cultured in T300 flasks until reaching a density of 8 × 10^5^ cells/ml. Cells were then centrifuged at 1000 rpm for 5 min, and the medium was replaced with fresh THP-1 complete medium supplemented with 75 ng/ml PMA. The cells were allowed to differentiate and adhere to the flask walls for 72 h at 37 °C in a 5% CO_2_ atmosphere. Post-differentiation, the medium was discarded, and cells were washed and replenished with RPMI-1640 medium. Half of the flasks (T300 × 3) continued incubation for 48 h, after which the supernatant was collected. The remaining flasks were treated with a uniform *C. albicans* suspension and incubated for 2 h at 37 °C, 5% CO_2_. Post-incubation, the flasks were washed thrice with sterile PBS to remove external *C. albicans*. Serum-free 1640 medium was then added, and the supernatant was collected after a further 6 h incubation. The collected supernatant was centrifuged at 3000 g for 10 min, and the resulting supernatant was stored on ice (or at − 80 °C if not used immediately, then thawed at 37 °C and placed on ice).

Exosome extraction was performed through differential centrifugation: The sample was first melted at 37 °C and then centrifuged at 2000 g for 30 min at 4 °C. The supernatant was transferred to a new tube and further centrifuged at 10,000 g for 45 min at 4 °C to remove larger vesicles. It was then filtered through a 0.45 μm filter membrane. The filtrate was ultracentrifuged at 100,000 g for 70 min at 4 °C. The pellet was resuspended in 10 mL of pre-cooled PBS and ultracentrifuged again under the same conditions. Finally, the pellet was resuspended in 300μL of pre-cooled PBS for storage at − 80 °C.

Transmission electron microscopy (TEM) was used to observe the exosome samples^53^: 10 μL of the exosome suspension was placed on a copper grid, allowed to settle for 1 min, and excess liquid was removed. After drying at room temperature for several minutes, the samples were observed under TEM at 100 kV.

Particle tracking analysis (NTA) of exosome samples^54^: frozen samples were taken, thawed in a 25 °C-water bath and placed on ice. Exosome samples were then diluted with 1 × PBS for direct NTA assay.

Western Blotting was performed to detect exosomal surface markers^51^: RIPA buffer (Beyotime, China), supplemented with protease and phosphatase inhibitors (Selleck, China), was used for protein extraction. Protein concentrations were determined using a BCA kit (Beyotime, China). Following protein blotting, detection and imaging were performed using a gel imager (Pinghao, Beijing, China) and chemiluminescent horseradish peroxidase substrate BrightTM ECL (Beyotime, China). To enhance specificity and clarity of detection, WB membranes were carefully trimmed to remove non-targeted areas before incubation with specific antibodies. This procedure was aimed at minimizing non-specific signals and focusing on the proteins of interest.

### PBA treatment of exosomal samples and high-throughput sequencing

The exosomes secreted by macrophages and those post-*C. albicans* infection were analyzed using PBA^18^. This involved the use of antibodies labeled with DNA probes containing a unique protein tag for protein identification, a molecular tag for repeat assays, and a universal binding site for subsequent processes. EV capture was performed using 96-well plates coated with Cholera Toxin subunit B (CTB)^55–58^. Antibody-tagged oligonucleotides on the same EV were assigned a unique EV tag. DNA sequences comprising EV tag-protein tag-molecular tag barcodes were prepared into sequencing libraries. Sequencing was conducted using the DNBSEQ-T7 platform (UWI, Shenzhen, China) or NovaSeq S4 (Illumina, USA). Raw sequencing data were converted from bcl to fastq files using MegaBolt (MGI) or bcl2fastq (Illumina).

### Exosome proteomic data analysis and statistical analysis^59–61^

Sequencing reads were analyzed using EVisualizer® decoding software (version 1.0, Secretech, Shenzhen, China) to generate EV ID-protein expression datasets as PBA raw data, and then analyzed for protein expression, combinations, and EV subgroups. To analyze the differentially expressed proteins (DEP) and differentially expressed protein combinations (DEPC), the following statistical analyses were performed, and the normality of the data was checked using the Shapiro–Wilk test. Homogeneity of the data was checked using F-test or Bartlett's test. When comparing two sets of data, the t-test was used if the data were normally distributed with a chi-squared variance. If the data are not normally distributed, the Mann–Whitney U test is used. Welch't test is used for data that is normally distributed but not chi-square. When comparing more than two groups (> 2 variables or categories), we chose the ANOVA test for normally distributed and chi-squared data sets. Significantly different expressions were analyzed by Duncan's test. If the data did not obey normal distribution or variance chi-square, the nonparametric test Kruskal–Wallis test was used instead. Significantly different expressions were analyzed using the paired Wilcoxon rank sum test. We utilized the Benjamini-Hochberg (BH) method to adjust p values. To generate EV subpopulations, the unsupervised FlowSOM^62^ algorithm is used. Distributed Stochastic Neighborhood Embedding (t-SNE) and Uniform Flow Approximation and Projection (UMAP) methods^63^ can be used to map the EV subpopulations. EV analysis based on the generated EV subpopulations is performed in the interactive interface of the software EVisualizer® online viewer (version 1.0, Secretech, Shenzhen, China).

### The strength and limitations of the present study

Exosomes, as a major hot topic at present, have been relatively little studied in the field of fungi, this paper seizes this breakthrough point and explores the immune role of macrophage-derived exosomes in fungal infections before and after *Candida albicans* infection; In this study, the proteomic resolution of single exosomes was carried out by this technology of PBA, which changed the dilemma of traditional exosome technology limited to total protein and total nucleic acid detection, and improved the precision of exosome protein detection to the level of single molecule, and realized the study of heterogeneity of exosomes with high precision and high sensitivity. The limitations of this study are that the inhibition of *Candida albicans* morphological transformation by macrophage-derived exosomes and the screening of possible biomarkers were explored and discussed based on the in vitro level, and future multicenter studies in animal models as well as in patient populations are needed to validate some of the early diagnostic markers and therapeutic targets and to confirm the functional roles of the above proteins.

### Supplementary Information

Supplementary Information.

## Data Availability

The datasets generated during and/or analysed during the current study are available from the corresponding author on reasonable request.
