# Supplementary figures and images for: Macrophage-derived CD36 + exosome subpopulations as novel biomarkers of Candida albicans infection
Source: Sci Rep. 2024 Jun 26;14:14723. doi: 10.1038/s41598-024-60032-7 (PMC11208550; doi:10.1038/s41598-024-60032-7)

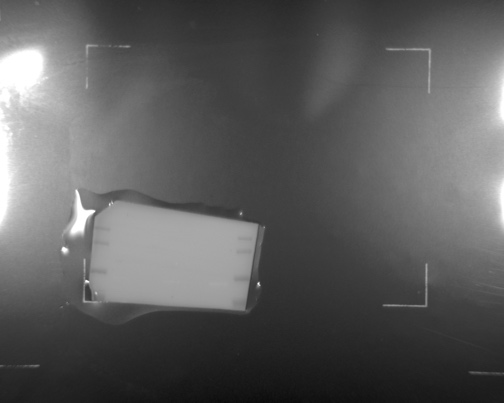

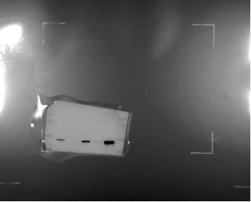


Calnexin BF calnexin MERGE


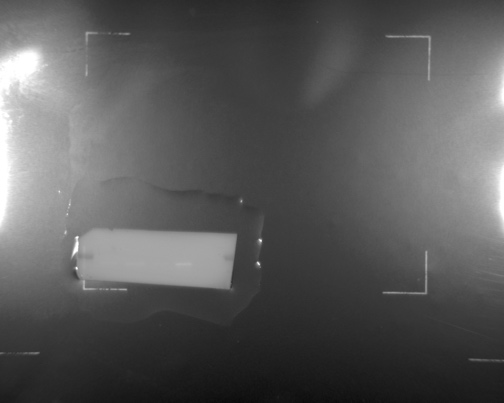

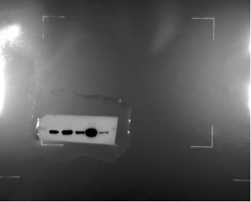


TSG101 BF TSG101 MERGE

Supplement: Supplementary file 1 — Supplementary Information. [file 41598_2024_60032_MOESM1_ESM.docx]
